# Speaking Clearly for the Blind: Acoustic and Articulatory Correlates of Speaking Conditions in Sighted and Congenitally Blind Speakers

**DOI:** 10.1371/journal.pone.0160088

**Published:** 2016-09-19

**Authors:** Lucie Ménard, Pamela Trudeau-Fisette, Dominique Côté, Christine Turgeon

**Affiliations:** Laboratoire de Phonétique, Université du Québec à Montréal, Center For Research on Brain, Language, and Music, Montreal, Quebec, Canada; IRCCS Istituto Auxologico Italiano, ITALY

## Abstract

Compared to conversational speech, clear speech is produced with longer vowel duration, greater intensity, increased contrasts between vowel categories, and decreased dispersion within vowel categories. Those acoustic correlates are produced by larger movements of the orofacial articulators, including visible (lips) and invisible (tongue) articulators. Thus, clear speech provides the listener with audible and visual cues that are used to increase the overall intelligibility of speech produced by the speaker. It is unclear how those cues are produced by visually impaired speakers who never had access to vision. In this paper, we investigate the acoustic and articulatory correlates of vowels in clear versus conversational speech, and in sighted and congenitally blind speakers. Participants were recorded using electroarticulography while producing multiple repetitions of the ten Quebec French oral vowels in carrier sentences in both speaking conditions. Articulatory variables (lip, jaw, and tongue positions) as well as acoustic variables (contrasts between vowels, within-vowel dispersion, pitch, duration, and intensity) were measured. Lip movements were larger when going from conversational to clear speech in sighted speakers only. On the other hand, tongue movements were affected to a larger extent in blind speakers compared to their sighted peers. These findings confirm that vision plays an important role in the maintenance of speech intelligibility.

## Introduction

### Clear speech versus conversational speech

Speech production can be thought of as a trade-off between two competing constraints: the need to ensure intelligibility and the tendency to expend minimal effort [1–3]. When required to speak clearly, speakers put more weight on intelligibility requirements [4–9]. Indeed, clear speech is defined as “a speaking style often adopted by talkers when speaking in difficult communication situations, e.g., when speaking in a very noisy or reverberant environment, or when talking to a hearing-impaired person. A clear speaking style may also be elicited by explicit instructions to a talker to produce highly enunciated speech.” (Uchanski, 2005: 207–208 [10]). Varying the speaking style substantially affects acoustic and articulatory characteristics of vowels and consonants. At the acoustic level, compared to conversational speech, clear speech is characterized by longer sound segments, tighter clustering within vowel categories in the acoustic space, expanded vowel spaces, and greater voice onset time (VOT) contrasts [11–16]. Tasko and Greilik [17] studied articulatory movements and acoustic characteristics of the word "combine" in 49 speakers from the University of Wisconsin X-Ray Microbeam Speech Production database [18]. They found that when speakers went from conversational speech to clear speech, they significantly increased vowel duration in the /aI/ diphthong. The speakers also significantly increased tongue movements and mandible movements. No significant gender differences were found. However, some studies found that the extent to which speech production is affected by the speaking condition varies across speakers. At the articulatory level, Perkell et al. [19] showed that when seven speakers were asked to produce clear speech, three speakers used larger and longer articulatory movements than in conversational speech; three others only increased vowel duration; and one speaker only increased root mean square (RMS) amplitude (an average measure of the intensity of a sound wave over time).

Despite inter-individual variability concerning the effects of speaking condition, there is consensus around the fact that when speakers switch from conversational speech to clear speech, they provide acoustic and articulatory cues that enhance speech intelligibility in listeners [8, 9, 20–23]. Gagné et al. [24] provided evidence that those cues are both audible and visible. Monosyllables and disyllables that were uttered by six speakers in clear and conversational speech were presented to 12 listeners in three conditions: audio only (only the sound was presented), visual only (only the speaker’s face was visible), and audiovisual (the speaker’s voice and face were presented). Even though speakers had different intelligibility scores (confirming that the correlates of produced clear speech vary across speakers), overall, in the three modalities, intelligibility scores were higher when syllables were produced in clear speech than when they were produced in conversational speech. More specifically, the clear speech effects (defined as the difference between the mean intelligibility score in clear speech versus the mean intelligibility score in conversational speech) among the six talkers varied from 4.51% to 25.17% in the auditory modality, from 2.49% to 15.57% in the visual modality, and from 2.08% to 14.76% in the audiovisual modality. These findings differ somewhat from those of Helfer [20], who reported that the increase in overall intelligibility in clear speech is 17% on average, and does not differ between modality (audio alone or audiovisual). Differences in the presentation of stimuli likely account for these discrepancies.

### Speech production in congenitally blind individuals

The fact that the acoustic and articulatory correlates of clear speech increase speech intelligibility in various sensory modalities (audio, visual, and audiovisual) suggests that some articulatory movements (e.g., of the lips) might be driven by auditory and visual perceptual requirements. In order to determine the effects of vision on speech articulation, we have been investigating the relationship between speech production and perception in individuals with congenital blindness. Although many studies have suggested that blind speakers have better auditory discriminatory abilities than sighted speakers in several tasks [25–28], very little is known about the effects of blindness on speech production in adults. In a recent study [28], we conducted acoustic analyses of isolated vowels produced by 12 congenitally blind adults and 12 sighted adults. Although the blind speakers demonstrated superior auditory discrimination for two contrasts, the sighted speakers produced significantly higher inter-vowel distances than the blind speakers for all five contrasts. In a follow-up study [29], we showed that the lack of visual cues resulting from congenital blindness significantly influences the articulatory strategies used by speakers to produce speech targets. Indeed, blind speakers used smaller differences in lip protrusion but larger differences in tongue position and shape than their sighted peers to produce French phonemes. To further investigate the relationship between the perceptual relevance of those articulatory strategies, we studied the production of French vowels under contrastive prosodic focus, a condition that is known to enhance the distinctiveness of phonemes [30]. According to Hay et al. [31] (2006), the different strategies used to increase perceptual saliency depend on the phonological contrasts of a language and on the different weights given to possible phonetic realizations of a contrast. We hypothesized that if the weighting given to gestures in French for an associated visual component (such as lip protrusion) was greater in sighted speakers than in congenitally blind speakers, those gestures could be used in different ways to signal focus in these two speaker groups. We found that, compared to the neutral prosodic condition, vowels produced under focus involved larger displacements of the lips for sighted speakers than for blind speakers. In contrast, sighted speakers had reduced displacements of the tongue when going from the neutral to the focused conditions, whereas blind speakers had significantly larger differences in tongue displacements for the two prosodic conditions. Thus, these studies suggest that there is a trade-off between displacements of the lips (visible articulators) and of the tongue (invisible articulator), which is regulated by vision. These results are likely the manifestation of additional constraints provided by vision on the links between articulatory movements and acoustic output in sighted individuals. Since blind speakers cannot see lip shape and jaw movement, they have more freedom to choose the articulators (lips, jaw, and tongue) to be recruited to achieve an intended acoustic product.

In the current paper, we investigate another perceptual enhancement condition, namely clear speech, in sighted and congenitally blind adult speakers of French. This condition is known to be driven by global intelligibility demands (on the domain of the entire speech sequences) as opposed to contrastive focus, in which intelligibility demands are increased locally (on a specific word, for instance). We had four hypotheses: 1) compared to conversational speech, speech produced in the clear speech condition would be associated with longer duration, larger acoustic contrasts between vowels, and tighter acoustic clustering within vowel categories; 2) at the articulatory level, clear speech would be produced by larger tongue, lip, and jaw displacements compared to conversational speech; 3) sighted speakers would increase visible lip gestures to a greater extent than blind speakers when going from conversational to clear speech; and 4) tongue displacement would be larger in blind speakers than in sighted speakers in clear speech compared to conversational speech.

## Materials and Methods

To test our four hypotheses, we conducted a speech production experiment with blind and sighted French speaking adults, using electromagnetometry. This research was approved by the Université du Quebec a Montreal's institutional review board (no 2012-05-4.3). All participants gave written, informed consent in accordance with the Board of Ethics of the University of Quebec in Montréal (UQAM).

### Participants

Twenty adults who participated in our previous studies were recruited [28–30]. Those studies were a few months apart and did not involve the same tasks as in the current experiment. Thus, the participants’ experience in previous speech studies could not have influenced the current results. Ten congenitally blind adults (five males and five females) and 10 sighted adult control participants (six males and four females) participated in the study. All speakers were native speakers of Canadian French living in the Montreal area. The blind speakers had a congenital, complete visual impairment, classified as class 3, 4, or 5 in the International Disease Classification of the World Health Organization (WHO). They had never had any visual perception of light or movement. They ranged in age from 26 to 52 years old (mean age, 44). They did not report any language disorders or motor deficits. They were initially selected from the *Institut Nazareth et Louis Braille*, a Rehabilitation Center in the Montreal area providing services to blind individuals. Table 1 presents the pertinent characteristics of the blind speakers. All control participants had perfect (20/20) vision or impaired vision corrected by lenses, resulting in near-perfect vision. They were 22 to 39 years old (mean age, 33). Despite the mean age difference between the sighted group and the blind group, it is highly unlikely that age could have influenced the acoustic and articulatory values investigated in the current study. For example, in two studies focusing on formant values in young and old speakers ([20, 22, 23, 32–33]), the elderly participants were older than 60 years and 69 years, respectively, which was much older than the speakers in the current study. All participants passed a 20-decibel hearing level (dB HL) pure-tone audiometric screening procedure at 500, 1000, 2000, and 4000 hertz (Hz). None of the participants had a learning disability or other known medical conditions.

**Table 1:** Characteristics of the 11 Blind Speakers/

Subject	Gender	Age	Etiology of blindness	Vision at birth	Current vision
S1B	F	48	retinitis pigmentosa	U	R.E. = 3/210
					L.E. = 0
S2B	F	40	congenital cataract	U	R.E. = 0
					L.E. = 6/1260
S3B	F	26	U	U	U
					(total blindness)
S4B	M	52	optic atrophy	total blindness	R.E. = 0
					L.E. = 0
S5B	M	40	detachment of the retina	U	R.E. = 2/180
					L.E. = 2/105
S6B	M	42	congenital cataract and congenital glaucoma	U	U
					(total blindness)
S7B	F	51	retinitis pigmentosa	total blindness	R.E. = 2/400
					L.E. = 2/400
S8B	F	45	congenital cataract	total blindness	U
					(total blindness)
S9B	M	42	congenital glaucoma	U	R.E. = 2/180
					L.E. = 3/180
S10B	F	45	congenital cataract	total blindness	R.E. = 0
					L.E. = 0

L.E. = left eye; R. E. = right eye; U = undetermined.

### Experimental procedure

The corpus consisted of ten repetitions of the Quebec French oral vowels /i y u e Ο o ε œ a/, embedded in the carrier sentence *Le mot /pVp/ me plaît* ("I like the word /pVp"). Stimuli were randomized across vowels. Speakers were asked to produce ten repetitions of the carrier sentence in two speaking conditions—clear speech and normal speech—according to the procedure described by Ménard et al. [12]. Normal speech was elicited by asking the subjects to pronounce the utterances at a conversational rate. Clear speech was elicited by asking the subjects to pronounce the words carefully without increasing loudness, since speaking loudly can introduce spectral changes (as described by Pickett et al. [34]). This clear-speech elicitation method is similar to the one used in Ménard et al. [12] in a study of speaking condition in deaf speakers with cochlear implants.

Acoustic and articulatory recordings were made using an electromagnetic articulograph (EMA) AG500 system (Linux version) using a sampling rate of 200 Hz in a soundproof room in the phonetics laboratory at the Université du Québec à Montréal [35]. During the recordings, the subjects were seated, with their heads within the EMA recording unit. The acoustic signal was recorded simultaneously with a Sony ECM-T6 microphone and digitized at 44,100 Hz using a Delta 1010 LT sound card. Calibration of the EMA system (see Carstens [35]) was performed before each recording. The obtained RMS values were all smaller than 7.53 with a mean value of 1.97. Eight sensors were attached to the upper and lower lip (at the vermillion line), lower incisor (at the gum limit) and on the tongue midline (tongue body, tongue blade, and tongue tip). The tongue tip sensor was placed 1 cm back from actual tongue tip in an attempt to minimize speech perturbation. The tongue body sensor was as far back as possible, and the tongue blade sensor was placed at a middle distance from the two other sensors. Four additional sensors were attached to the left and right mastoids and on the left and right lateral upper incisors at the gum limit and were used for head-movement correction. After the recording, the position (x: back/front, y: left/right, and z: high/low) and orientation (phi: azimuth and theta: elevation) of each sensor through time was extracted using the Linux version of the EMA software (Carstens CalcPos). Sensor positions and orientation were corrected for head movements using a Matlab procedure developed by Mark Tiede (Haskins Laboratory, New Haven, CT), which uses the upper incisor sensor (left or right) and the mastoid sensor (left or right) that shows the least distortion (smaller standard deviation in terms of Euclidean distance to the three other reference sensors). All values were translated and rotated to this reference frame.

### Data analysis

For each target vowel, acoustic and articulatory measures were extracted. First, acoustic signals were down-sampled to 22050 Hz, after low-pass filtering (cut-off frequency of 10000 Hz). The first three formant frequencies were then estimated for each vowel, using the linear predictive coding (LPC) algorithm implemented in the Praat speech analysis program. The number of poles varied from 12 to 18. A 14-ms Hamming window centered at the vowel mid-point was used, with a pre-emphasis factor of 0.98 (pre-emphasis from 50 Hz for a sampling frequency of 22050 Hz). Formant measurement errors were detected by comparing, for each vowel, the automatically extracted formant values overlaid on both a wide-band spectrogram and on a fast Fourier transform (FFT) cross-spectrum obtained using the same Hamming window. The formant frequencies were then converted to the mel scale (since this scale approximates the ear’s integration of frequency), according to the formula F_mel_ = 550*ln(1+F_Hz_ / 550). The produced stimuli were represented in the traditional F1 vs. F2 vs. F3 space, in mels. This three-dimensional space was used rather than the F1 vs. F2 space to account for possible shifts in formant-cavity affiliations across subjects, which yield greater contrast between two vowel categories in the F2 vs. F3 space than in the F1 vs. F2 space. This is the case, for example, in the /i/ vs. /y/ rounding contrast in French [36]. Fundamental frequency (F0) measurements were made using the autocorrelation method. Vowel intensity (in dB sound pressure level [SPL]) was also extracted.

In the acoustic space, two measures of dispersion were calculated. First, Average Vowel Space (AVS) is defined as the average values of Euclidean distances, in a two-dimensional or three-dimensional space, between vowel categories [12, 37]. In the current study, the three-dimensional acoustic space F1 vs. F2 vs. F3 (in mels) was considered. More specifically, for each speaker and each possible vowel pair, Euclidean distances were calculated between the vowel loci determined by mean, mel-transformed formant frequencies F1, F2, and F3. In order to calculate dispersion values for each vowel type (second measure of dispersion), the Euclidean distance of each token from the mean position of all tokens of that phoneme was determined in the formant space. Those distances were then averaged across repetitions to obtain the dispersion measure for that vowel.

At the articulatory level, sensor positions were extracted at the vowel midpoint. Articulatory measures included x (front–back) and z (low–high) positions of the upper lip, lower lip, jaw, tongue tip, tongue blade, and tongue body. For each vowel, the mean positions of the upper lip, lower lip, tongue tip, tongue blade, and tongue dorsum sensors in the normal-speech condition and in the clear-speech condition were computed. Contrast distances (AVS) between all possible pairs of vowels were then calculated in the two-dimensional articulatory space corresponding to each sensor’s front-back and high-low position. For each speaker, average values of all the Euclidean distances were obtained. This procedure was done in each speaking condition.

A measure of within-category vowel dispersion was also calculated. For a given vowel category, the Euclidean distances between each repetition of this vowel and the mean sensor position of all repetitions of that vowel category were computed. The mean value of those Euclidean distances corresponded to the within-category dispersion for that vowel. Vowel dispersion values for all ten vowels under study were then averaged for each speaking condition and speaker group.

Repeated measures multiple analyses of variance (MANOVA) were conducted (following Ménard et al., 2004), with the subject group and the speaking condition as the independent variables. One MANOVA was conducted with the following acoustic measures as the dependent variables: F0, intensity, and duration. Another MANOVA was conducted on the articulatory values, and involved six dependent variables: Euclidean distances in the upper lip space, the lower lip space, the jaw space, the tongue tip space, the tongue blade space, and the tongue back space. Finally, a repeated-measure ANOVA was conducted on AVS in the formant space. Violations of sphericity were checked through the Greenhouse-Geisser epsilon variable. Since no violation was found, the original degrees of freedom were reported. Effect sizes, corresponding to eta-squared values, and values of Wilk’s lambda are reported. To evaluate the extent to which trading relationships were involved between acoustic parameters (F0, intensity, duration, AVS in the formant space) and lip, jaw, and tongue position, multiple linear regression analyses were carried out for each speaker group. Only the analysis yielding significant results will be presented.

## Results

### Acoustic results

**Fig 1:**
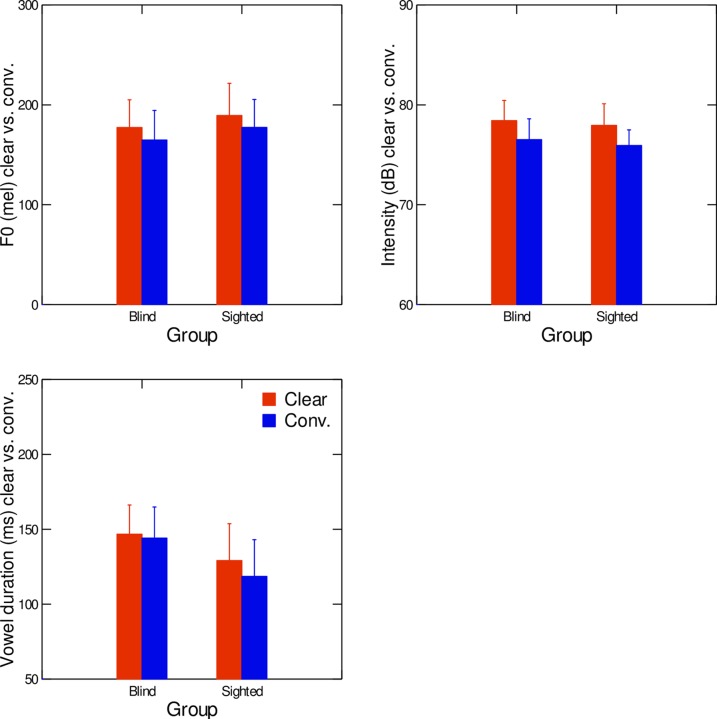
Average values of F0 (upper left graph), intensity (upper right graph), and duration (lower graph) in clear (red) and conversational (blue) speech, for both sighted and blind speakers. Error bars are standard errors.

Average values of vowel duration, vowel intensity, and F0 in the clear-speech and conversational-speech conditions, for blind and sighted speakers, are shown in Fig 1. Data were averaged across vowels. At the multivariate level, a significant main effect of speaker group was found (F(3,18) = 7.33, *p* < .01, Wilks' lambda = .450, η^2^ = 0.55), as well as a significant main effect of speaking condition (F(3,18) = 6.84, *p* < .01, Wilks' lambda = .467, η^2^ = 0.53). The interaction between the speaking condition and the group was not significant (F(3,18) = 0.38, *p* > .05, Wilks' lambda = .940, η^2^ = 0.06). Results of univariate analyses conducted on F0 values revealed a significant effect of speaking condition, with increased F0 in the clear-speech condition compared to the conversational speech condition (F(1,20) = 12.19; p<0.01). No significant effect of the interaction of speaking condition and speaker group was found (F(1,20) = 1.93; p>0.05). Looking at vowel intensity values, univariate results for the data depicted in Fig 1 (upper right panel) across speaker groups and speaking conditions did not reveal any significant effect of speaking condition as a main effect (F(1,20) = 0.77, *p >* .05) or in interaction with speaker group (F(1,20) = 0.01, *p >* .05). Regarding average vowel duration (Fig 1, lower panel), univariate results did not suggest any significant effect of speaking condition on vowel duration (F(1,20) = 1.23, *p >* .05), but blind speakers had significantly longer vowel duration than their sighted peers (F(1,20) = 18.23, *p <* 0.01).

This pattern was also found in our previous study [30]. No significant effect of interaction between the independent variables was found (F(1,20) = 1.26, *p >* .05).

**Fig 2:**
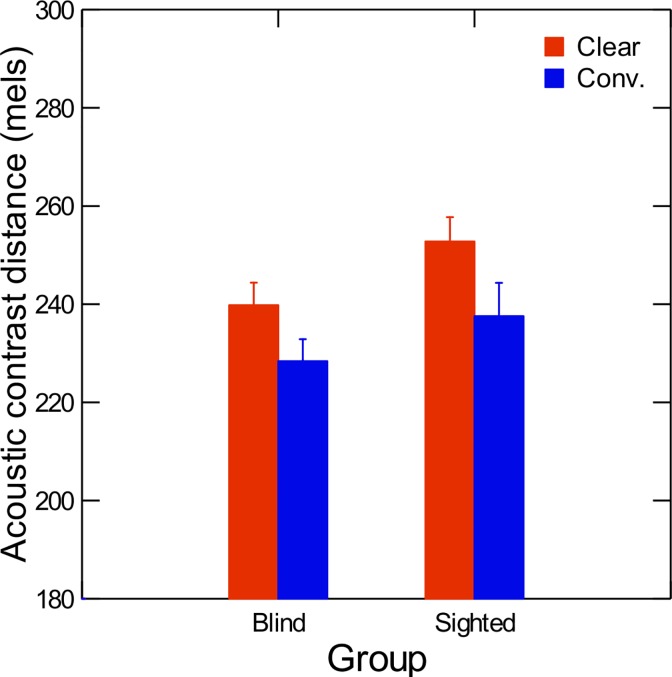
Average values of acoustic contrast distances in clear (red) and conversational (blue) speech, for both sighted and blind speakers. Error bars are standard errors.

Average contrast distances between vowel categories in the acoustic F1 vs. F2 vs. F3 space (in mels), are shown in Fig 2, for each speaker group and for each speaking condition. Results of a repeated-measures ANOVA with speech condition (clear or conversational) as the within-subject factor and participant group (blind or sighted) as the between-subject factor revealed a significant effect of speaker group on contrast distances among vowels (F(1,18) = 12.76; p<0.05; η^2^ = 0.99). Pooling the data across speaking conditions showed that sighted speakers produced vowels that were spaced further apart in the acoustic space than their blind peers. Furthermore, the analysis showed a significant main effect of speaking condition on contrast distance (F(1,18) = 20.85; p<0.001; η^2^ = 0.53) with vowels produced in clear speech being more contrasted than vowels produced in conversational speech. No significant effect of the interaction between speaker group and speaking condition was found ((F(1,18) = 0.08; p>.05)

**Fig 3:**
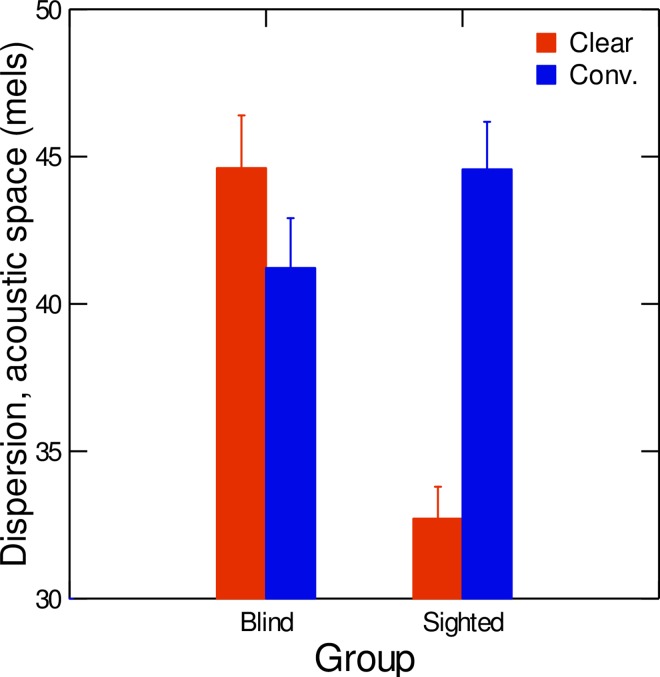
Average values of within-category vowel dispersion in clear (red) and conversational (blue) speech, for both sighted and blind speakers. Error bars are standard errors.

Regarding within-category vowel dispersion, average data are displayed in Fig 3. Results of the repeated measures ANOVA conducted on the average dispersion values in the acoustic F1 vs. F2 vs. F3 space revealed a significant effect of speaker group, with blind speakers having overall larger within-category vowel dispersion than sighted speakers (F(1,18) = 9.14;p<0.01; η^2^ = 0.31). Furthermore, the interaction of speaker group with speaking condition was significant (F(1,18) = 12.72; p<0.01; η^2^ = 0.82). Post-hoc tests showed that in sighted speakers only, vowels were significantly more tightly clustered within categories in the clear-speech condition compared with the conversational-speech condition (F(1,18) = 15.00; p<0.001).

### Articulatory results

**Fig 4:**
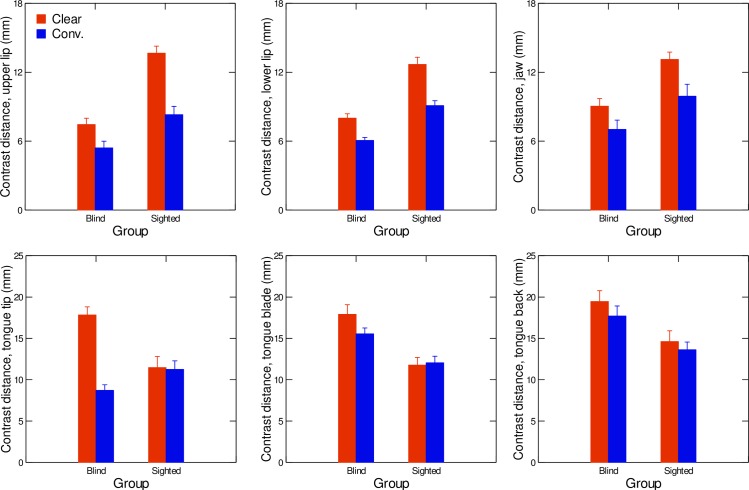
Average values of articulatory contrast distances in clear (red) and conversational (blue) speech, for both sighted and blind speakers in the upper lip space (upper left graph), lower lip space (upper middle graph), jaw space (upper right graph), tongue tip space (lower left graph), tongue blade space (lower middle graph), and tongue back space (lower right graph). Error bars are standard errors.

The articulatory strategies exploited by both participant groups were investigated through a detailed analysis of articulatory positions. The average contrast distances between vowel categories were calculated for each of the six sensors (upper lip, lower lip, jaw, tongue tip, tongue blade, and tongue dorsum) in the two-dimensional spaces corresponding to the sensor’s x (front-back) and z (high-low) dimensions. Data are shown separately for each speaker group (blind or sighted) and for each speaking condition (clear or conversational) in Fig 4. At the multivariate level, MANOVA results revealed that the speaker group had a significant effect on the articulatory data (F(6,15) = 20.42, *p* < .001, Wilks' lambda = .109; η^2^ = 0.89), as did the speaking condition, (F(6,15) = 13.98, *p* < .01, Wilks' lambda = .151; η^2^ = 0.85). The group factor significantly interacted with the speaking condition, (F(6,15) = 7.43, *p* < .01, Wilks' lambda = .252; η^2^ = 0.75).

For the upper lip sensor (Fig 4, upper left graph), univariate results revealed a significant main effect of speaking condition (F(1,18) = 30.87; p<0.001), with contrasts being significantly larger in the clear-speech condition than in the conversational-speech condition. Blind speakers produced significantly smaller upper lip contrasts than sighted speakers (F(1,18) = 49.07; p<0.001). The MANOVA also suggests a significant interaction between speaker group and speaking condition (F(1,18) = 14.69; p<0.01). The contrast difference between the clear and conversational conditions was larger in sighted speakers than in blind speakers (F(1,18) = 10.93; p<0.05). The lower lip sensor (Fig 4, upper mid graph) displayed a similar pattern of results. Specifically, blind speakers produced significantly smaller contrasts than their sighted peers (main effect of speaker group (F(1,18) = 11.375; p<0.01)) and clear speech was produced with significantly larger lower lip contrasts than conversational speech (main effect of speaking condition (F(1,18) = 22.42; p<0.01)). The interaction between speaker group and speaking condition was also significant (F(1,16) = 10.52; p<0.05), with blind speakers producing smaller contrast differences between conversational and clear speech compared to the sighted speakers. Contrasts in terms of jaw positions (Fig 4, upper right graph) revealed a main effect of speaker group (F(1,18) = 14.99; p < .01)): blind speakers produced smaller jaw contrasts than sighted speakers. The effect of speaking condition was also significant (F(1,18) = 11.34; p<0.01), with clear speech involving significantly larger jaw contrast distances than conversational speech. The interaction between speaker group and speaking condition was not significant (F(1,20) = 0.58; p>0.05).

The three tongue sensors had different patterns of results. First, univariate results of the MANOVA for the tongue tip variable (Fig 4, lower left graph) revealed a significant interaction of speaker group and speaking condition (F(1,18) = 20.94; p<0.01). As shown in Fig 4, increased values of contrast distances are found when going from conversational to clear speech for blind speakers only. As for contrast distances in the tongue blade articulatory space (Fig 4, lower mid graph), a significant effect of speaker group was found, with blind speakers producing larger tongue blade contrast distances than sighted speakers (F(1,18) = 24.54; p<0.01). No significant effect of speaking condition as a main effect (F(1,18) = 1.74; p<0.05) or in interaction with speaker group (F(1,18)-2.77; p<0.01) was found. Finally, contrast distances in the tongue back articulatory space (Fig 4, lower right graph) were significantly larger for blind than for sighted speakers (F(1,18) = 60.02; p<0.01). Speaking condition did not have a significant effect, either as a main effect (F(1,18) = 2.31; p>0.05) or in interaction with speaker group (F(1,18) = 0.18; p>0.05).

**Fig 5:**
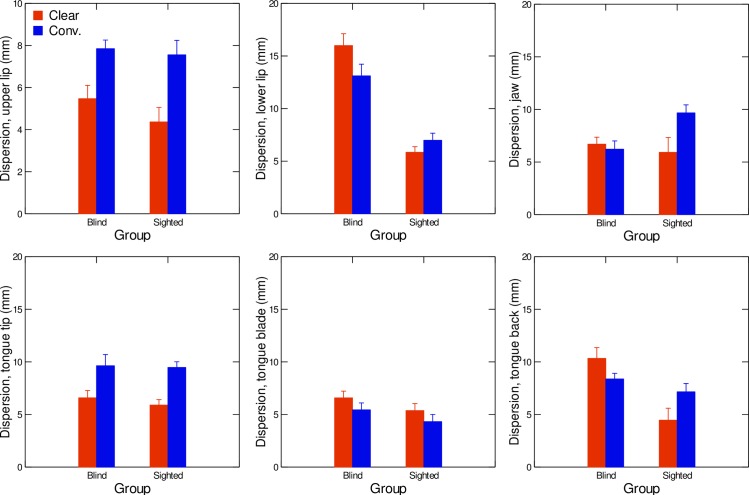
Average values of within-category vowel dispersion in clear (red) and conversational (blue) speech, for both sighted and blind speakers in the upper lip space (upper left graph), lower lip space (upper middle graph), jaw space (upper right graph), tongue tip space (lower left graph), tongue blade space (lower middle graph), and tongue back space (lower right graph). Error bars are standard errors.

Average values of within-category vowel dispersion in each sensor’s articulatory space are shown in Fig 5. A repeated measures MANOVA was conducted with vowel dispersion for each of the six sensors as the dependent variables, speaker group as the between-subject factor, and speaking condition as the within-subject factor. At the multivariate level, the analysis showed a significant main effect of speaker group (F(6,13) = 5.93, *p* < .01, Wilks' lambda = .268 (η^2^ = 0.73)). The multivariate results also revealed a significant effect of speaking condition (F(6,13) = 14.85, *p* < .001, Wilks' lambda = .127 (η^2^ = 0.87)) and of the interaction between speaker group and speaking condition on the dispersion values (F(6,13) = 14.56, *p* < .001, Wilks' lambda = .130 (η^2^ = 0.87)). At the univariate level, for the upper lip sensor (Fig 5, upper left panel), dispersion values did not differ significantly according to speaker group (F(1,18) = 0.97; p>.05), but they were significantly higher in the conversational condition than in the clear condition (F(1,18) = 35.00; p<0.001). For the lower lip sensor (Fig 5, upper mid panel), vowel dispersion was significantly larger in the blind group than in the sighted group (F(1,18) = 62.57; p<0.001). A significant interaction of speaking condition and speaker group was also observed (F(1,18) = 85.76; p<0.001), with blind speakers having larger values in the clear speech condition than in the conversational speech condition, whereas sighted speakers produced larger dispersion values in the conversational speech condition than in the clear speech condition. For the jaw sensor (Fig 5, upper right panel), univariate results did not reveal any significant effect of speaking condition (F(1,18) = 4.35; p>.05) or speaker group (F(1,18) = 1.13; p>.05). Concerning within-category dispersion of the tongue tip (Fig 5, lower left panel), values were significantly larger in conversational speech than in clear speech (F(1,18) = 19.40;p<0.01). However, no significant interaction effect between speaking condition and speaker group appeared (F(1,18) = 0.08;p>.05). As for the tongue blade sensor (Fig 5, lower mid panel), speaker group and speaking condition did not have a significant effect on dispersion values (speaker group: F(1,18) = 0.08; p>.05; speaking condition: F(1,18) = 3.13;p>.05). However, dispersion values in the tongue dorsum dimensions (Fig 5, lower right panel), varied significantly according to speaker group (F(1,18) = 22.56; p<0.01), with blind speakers having globally larger dispersion values than sighted speakers. A significant interaction between speaker group and speaking condition was found (F(1,18) = 10.48; p<0.01). More specifically, for blind speakers, dispersion values were larger in clear speech than in conversational speech, whereas the reverse pattern was found for sighted speakers (larger values in the conversational condition than in the clear condition).

Last, to investigate the extent to which articulatory contrasts are related to acoustic contrasts, multiple regression analyses were conducted for each speaker with average contrast distances in the formant space as the dependent variable and the following independent variables (predictors): contrast distances in the upper lip space, contrast distances in the jaw space, and contrast distances in the tongue back space. Those parameters are traditionally acknowledged as being the three main phonetic articulators involved in vowel production. Table 2 provides values of standardized beta weights for each speaker. A repeated-measure ANOVA conducted on those beta weights with subject group as the between-subject variable and articulatory space as the within-subject variable revealed a significant interaction between group and articulatory sensor (F(2,36) = 25.70; η^2^ = 0.59). Values of standardized beta weights, reflecting the weight of the articulatory parameter in the model predicting the acoustic contrast distance, were larger in blind speakers than in sighted speakers for the tongue sensor, whereas they were smaller for the upper lip sensor. No significant difference was observed for the jaw sensor.

**Table 2:** Standardized beta weights obtained from multiple regression analyses performed for each speaker with AVS in the formant space as the dependent variable and upper lip contrast distance (UL), jaw contrast distance (JAW), and tongue back contrast distance (BACK) as the predictors (independent variables).

Speaker	UL	JAW	BACK
B1	0.18	0.18	0.39
B2	0.21	0.13	0.23
B3	0.11	0.23	0.45
B4	0.25	0.17	0.23
B5	0.14	0.32	0.45
B6	0.23	0.42	0.52
B7	0.09	0.12	0.26
B8	0.17	0.19	0.42
B9	0.16	0.21	0.38
B10	0.27	0.32	0.28
S1	0.29	0.35	0.23
S2	0.31	0.28	0.21
S3	0.34	0.47	0.23
S4	0.35	0.23	0.19
S5	0.19	0.11	0.12
S6	0.38	0.28	0.11
S7	0.33	0.25	0.23
S8	0.26	0.27	0.21
S9	0.29	0.17	0.23
S10	0.32	0.19	0.15

## Discussion

To investigate the role of visual information in the production of clear speech, we examined acoustic and articulatory characteristics of vowels produced by congenitally blind and sighted speakers of French. Our first hypothesis was that acoustic differences in clear speech vs. conversational speech would be found in terms of duration, pitch, contrast distances, and within-category vowel dispersion. Our results partly confirm this hypothesis. Indeed, as shown in Figs 1 to 3, compared to conversational speech, vowels produced in clear speech were significantly longer, higher, and spaced further apart in the vowel space. However, tighter clustering within categories in clear speech was only found in the sighted speakers. These results agree with results from Ménard et al. [12], who studied contrast distances and vowel dispersion in speakers with cochlear implants.

At the articulatory level, we hypothesized that clear speech would be produced by larger tongue, lip, and jaw displacements compared to conversational speech (hypothesis 2). Again, this hypothesis was partly confirmed, in that a significant main effect of speaking condition was found for the contrast distances in terms of the upper lip, lower lip, and jaw.

However, in line with our third hypothesis, a significant effect of the interaction between speaking condition and speaker group was found for the two lip sensors: sighted speakers increased visible lip contrasts to a greater extent than blind speakers when going from conversational to clear speech. As for the tongue sensors, a significant interaction of speaking condition and speaker group was also found, but only for the tongue tip sensor, for which contrast distances increased from conversational speech to clear speech for the blind speakers only. Furthermore, values of standardized beta weights, reflecting the weight of the articulatory parameter in the regression model predicting the acoustic contrast distance, were larger in blind speakers than in sighted speakers for the tongue sensor, whereas they were smaller for the upper lip sensor. These results show that when congenitally blind speakers need to produce especially intelligible speech, such as in a clear-speaking condition, they use articulatory strategies that differ from their sighted peers. The fact that the blind speakers used the lingual articulator to a larger extent than the sighted speakers to enhance speech intelligibility in the clear-speech condition suggests that the tongue gesture is more robustly linked to vowel targets in blind speakers than in sighted speakers. Lip movements, on the other hand, are more weakly related to the phonemic target in blind speakers than in sighted speakers and thus are recruited to a lesser extent to enhance speech intelligibility. Previous studies aimed at investigating perceptual acuity in our cohort of blind speakers have ruled out the possibility of auditory acuity driving the pattern of variation found for dispersion and contrast values ([28]). Our results more likely suggest that congenital visual deprivation altered production-perception relationships in speech. The effects of such differences on the intelligibility of blind and sighted speakers are currently being examined through perceptual testing.

Concerning within-category articulatory dispersion, this variable was reduced in clear speech compared to conversational speech for the upper lip sensor (blind and sighted speakers), and the tongue tip sensor (sighted and blind speakers), and the tongue dorsum sensor (sighted speakers only). For two articulators (lower lip and tongue dorsum), blind speakers increased within-category vowel dispersion when going from conversational to clear speech. When results were pooled together, blind speakers had larger within-category dispersion in terms of lower lip and tongue dorsum compared with sighted speakers. This larger dispersion in congenitally blind speakers compared to their sighted peers suggests that phonetic variability related to vowel phonemes in French is greater in blind speakers. It has been suggested that increased speech variability is an index of poorly tuned motor control, as is the case in speech development ([38]) or in profoundly deaf speakers ([3, 12, 37]). According to Perkell’s work [3, 12], among others, within-category vowel dispersion represents the acoustic speech goal and varies depending on speaking condition (eg, vowel dispersion is reduced when going from conversational to clear speech) and auditory deprivation (eg, vowel dispersion is larger in deaf speakers wearing cochlear implants than in hearing speakers). In the present study, increased vowel dispersion for the blind suggests that the size of the acoustic/auditory region associated with a phonemic category is larger, a pattern of result that might be related to reduced precision in speech production, due to visual deprivation. Furthermore, several studies have shown perceptual somatosensory enhancement in blind speakers compared to sighted speakers (eg.: [39]). In postural control, it has been shown that proprioceptive acuity in enhanced in blind speakers compared to sighted speakers, but that this sensory advantage does not translate into increased postural control of the ankle ([40]). These results suggest that visual deprivation might alter the ability to control a motor target, as the data on dispersion suggest in our study. Further studies are currently in progress to test this hypothesis.

In our corpus, although vowel duration was not significantly different in different speaking conditions, when the data were averaged across speaking conditions, congenitally blind speakers produced longer vowels than their sighted peers, which should give them more time for larger movements. However, AVS values (in the acoustic and articulatory spaces) do not suggest that distance is increased (at least not for all articulators). We found similar results in our previous study of contrastive emphasis in French speakers [30]. Since blind speakers have been found to outperform sighted speakers in perceiving ultrafast speech ([41]), this pattern of results, at the production level, is surprising.The production of intelligible speech targets might require more time because the weight of the auditory (and possibly, somatosensory) components in the speech goal is larger in blind speakers than in sighted speakers. Further experiments are currently conducted in order to test this hypothesis and, more generally, to better understand the impact of visual deprivation on the nature of the phonological representations and in their phonetic implementation.
